# Adolescents’ and young people’s experiences of social relationships and health concerns during COVID-19

**DOI:** 10.1080/17482631.2023.2251236

**Published:** 2023-08-28

**Authors:** Annelie J Sundler, Disa Bergnehr, Sadiyya Haffejee, Humera Iqbal, Marjorie Faulstich Orellana, Ana Vergara Del Solar, Sophia L. Angeles, Charlotte Faircloth, Lu Liu, Anita Mwanda, Mauricio Sepúlveda Galeas, Thandi Simelane, Katherine Twamley, Laura Darcy

**Affiliations:** a Department of Caring Science, University of Borås, Borås, Sweden; b Department of Pedagogy and Learning, Linnaeus University, Växjö, Sweden; c Centre for Social Development in Africa, University of Johannesburg, Johannesburg, South Africa; d Thomas Coram Research Unit, University College London, London, UK; e School of Education and Information Studies, University of California, Los Angeles, USA; f Faculty of Humanities, Universidad de Santiago, Santiago, Chile; g College of Education, Penn State University, Pennsylvania, USA; h Faculty of Psychology, Universidad Diego Portales, Chile

**Keywords:** Experiences, pandemic, youth, qualitative research, thematic analysis

## Abstract

**Purpose:**

To illuminate the meaning of social relationships and health concerns as experienced by adolescents and young people during the COVID-19 pandemic.

**Methods:**

A longitudinal qualitative study was conducted. Data reported from 172 adolescents and young people aged 12–24 years in five countries; Chile, South Africa, Sweden, the United Kingdom and the United States collected from May 2020 to June 2021 were analysed via thematic analysis.

**Results:**

Adolescents and young peoples’ experiences of social relationships and health concerns were described in seven themes: Family proximity, conflicts and frustration; difficulties and challenges related to limited living space; peer relations and maintaining friendship in times of social distancing; the importance of school as a place for interaction; vulnerability, emotional distress and uncertainty about the future; health concerns and sense of caring for others; and worries and concerns related to financial hardship. These reports show that the changes to everyday life that were introduced by public responses to the pandemic generated feelings of loneliness, vulnerability, and emotional distress, as well as increased sense of togetherness with family.

**Conclusions:**

The everyday lives of adolescents and young people were restricted and affected more by the consequences of the pandemic than by the COVID-19 virus. These experiences had various impacts on well-being and mental health, where some individuals felt more exposed and vulnerable to emotional distress and loneliness than others. Family and peer relationships could be protective and support a sense of togetherness and belonging. Hence, social relationships are important to provide emotional support. Support for adolescents and young people should be tailored accordingly around social and emotional concerns, to encourage health and well-being.

## Introduction

The COVID-19 pandemic has had a global impact, with varying consequences to children’s and adolescent’s health and well-being (World Health Organization, 2022). Even if the pandemic was global, policies varied across nations. Still, education systems and essential health services were disrupted across nations, potentially having long-term impacts on children’s and adolescents’ health. For adolescents, the pandemic occurred at a stage of development between childhood and adulthood where patterns of behaviour that can support health are commonly established. There is limited in-depth knowledge about health concerns from children’s perspectives during the pandemic. This study was conducted to understand and give voice to adolescents’ and young people’s experiences of this period to thereby contribute to positive future development for all children and adolescents.

### Health, well-being and social relationships

Health is a resource for everyday life (WHO, 1986) and defined as “a state of complete physical, mental and social well-being and not merely the absence of disease or infirmity” (WHO, 1946, pg.1). Gadamer (1993/1996 states that health has a hidden character and may not actually present itself to us until something is missing, or disturbs our health, as for instance the COVID-19 pandemic. Moreover, health is closely related to experiences of well-being and our bodily being in a social context (Merleau-Ponty, 2002). Thus, health is not merely a physical attribute. Rather, health and our health concerns may be multifaceted: influenced by our social relationships, our capabilities (Sarvimäki, 2006) and balance in life (Lipworth et al., 2011). Lack of social relationships and loneliness can have consequences on our health and well-being and have been recognized as critical public health issues (Lim et al., 2020). Loneliness is known to influence children’s mental health and well-being negatively (Pearce et al., 2021; Qualter et al., 2013). Consequently, feelings of loneliness and isolation are considered risk factors for depression and mental ill health in children and adolescents (Lyyra et al., 2018).

### Children and young people’s perspectives on health and the pandemic

The pandemic has contracted the social worlds for many children into their home and the pandemic brought children’s health and well-being into scrutiny. A study on adolescent girls’ lived experiences of health prior to the pandemic described health as a phenomenon closely linked to their ability to manage everyday life, influenced by their social relationships. Experiences of health were described in terms of a mood of “being”, shaped and reshaped in everyday life (Larsson et al., 2013). Consequently, health is a phenomenon that unfolds in our everyday life. This perspective can help understand children’s experiences and health concerns during COVID-19.

Public and political concerns have been raised about the impact of the pandemic on children’s health, development and learning. Researchers have highlighted challenges to children’s well-being and social relationships (Katz et al., 2021; Kerekes et al., 2021; Kusumaningrum et al., 2022; Orgilés et al., 2020; Petrowski et al., 2021). The uncertainty that young people lived with, especially during the early months of the pandemic when little was known about the disease, along with feelings of social isolation, fear and anxiety that were engendered during the lock-down period, may have additional long-term impacts on children and adolescents’ health and well-being (Liu et al., 2020; Minds, 2020). Heightened rates of social isolation and feelings of loneliness were reported by young people during the pandemic, considered risk factors for mental health problems, depression, and mental ill health (Lee et al., 2020; Lisitsa et al., 2020; Loades et al., 2020). However, to the best of our knowledge, there are few studies that have illuminated possible benefits and experiences on how to make meaning from this period of forced isolation from peers.

Public health measures during the COVID-19 pandemic resulted in a reduction in social interaction outside the home. Research on adolescents in Morocco, Serbia, Sweden, US, and Vietnam suggests that such restrictions impacted both positively and negatively on children’s self-reported quality of life and well-being (Kerekes et al., 2021). Family members in Italy described experiences of increased frustration and irritation, although lockdowns could also strengthen family ties and lead to intensified and/or new care practices (Panarese & Azzarita, 2021). This ambiguity was also found in a study based in the United Kingdom, where increased closeness and feelings of isolation were reported. On one hand, children stated that they enjoyed spending more time with their family during lockdown and this strengthened their togetherness (Levita et al., 2020; McKinlay et al., 2022). On the other hand, children talked about feeling lonely and isolated from peers, and many stated that they struggled with a decline in their mental health (McKinlay et al., 2022). However, in Sweden, where there was no lockdown and schools remained open for most children, adolescents reported that they experienced more conflicts with parents than before the pandemic (Kapetanovic et al., 2021), indicating that it was potentially more than restrictions that impacted on familial relationships.

### Disruption of school, social isolation and loneliness

Schooling for children and adolescents varies globally, and much attention has gone to disruptions in academic learning based on school closures, while less consideration has been given to the social impacts. School is often a space where children spend much time with peers and disruption of school may be a source of loneliness. Research suggests that negative impacts of the pandemic during lockdown were seemingly alleviated through regular on-line contacts with peers (Cowie & Myers, 2020; Panarese & Azzarita, 2021). However, not all children had access to technology needed for on-line school or contacts with friends when ordinary school was disrupted (Tomlinson et al., 2021). Moreover, children and adolescents reported stress and anxiety during and after the COVID-19 pandemic (Meherali et al., 2021).

Given the potential for long-term consequences from the COVID-19 pandemic on adolescents and young people, it is important to have an in-depth understanding of their health, health concerns and social isolation during the pandemic. This study aimed to illuminate the meaning of social relationships and health concerns as experienced by adolescents and young people during COVID-19 pandemic.

## Materials and methods

### Study design

A longitudinal qualitative study combining interviews and diaries was conducted.

### Ontological and epistemological approach

In this qualitative study, we adopted a human science orientation with an existential view of being human based on the work of Merleau-Ponty (2002) to understand lived experiences and human phenomena such as health, health concerns and social relationships. This philosophical understanding builds on an interest in the lifeworld and human intersubjectivity and has implications for both data collection and analysis. We emphasize openness, meaning that researchers must be sensitive to the studied phenomenon. The ontological view builds on nondualist and relational assumptions about the nature of human beings and our subjectiveness, and includes the interconnectedness between mind, body, and spirit. Our subjectivity and who we are, our resources and skills, all depend on our social situations and relationships. This qualitative approach is appropriate when exploring subjective experiences of individuals, their thoughts and perceptions of the impact of COVID-19 on everyday life and on social relationships with family and friends.

### National settings

This study is part of the ICO-FACT project, an international Consortium on Family and Community in the Time of COVID-19 to investigate experiences of family and social life (Twamley et al., 2023; also see https://fact-covid.wixsite.com/study/i-cofact). Within ICO-FACT data were collected from May 2020 to June 2021, at the height of the COVID-19 pandemic and during a period in which many countries closed schools and restricted public interactions. The five countries represented in this study responded in different ways to the pandemic. Chile, South Africa and the UK (with some small regional differences) experienced intermittent national lockdowns, during which schools and other childcare institutions closed, along with other “non-essential” businesses and organizations. The UK and South Africa had “stay at home orders” with limited permissions to leave the house, except for essential work or necessities. Meanwhile, in Chile, the pandemic coincided with an economic crisis and social turbulence, which led the government to initiate a curfew that was in effect for more than 1.5 years. The US had a diversity of policies in place since recommendations were offered at the federal level but were taken up in different ways in different localities. Many schools closed in the spring of 2020 and again in the 2020–21 year, with continuing closings/re-openings in 2021–22. Sweden had no enforced lockdown, and used a strategy that was based on recommendations. Sweden is known for high levels of trust between people and authority (Holmberg & Rothstein, 2015). This was considered an advantage during the pandemic when the main recommendations were social distancing, working from home, handwashing, and staying home when ill or with any symptoms of a cold. Upper-secondary school students (16–19 years-old) experienced periods of on-line teaching, while preschools, primary and middle schools as well as organized leisure activities for adolescents 15 years old or younger, mainly continued as usual. Internationally, Sweden was considered to be an outlier in its handling of the pandemic.

### Sampling

Purposive sampling strategies were used by all five national research teams to recruit families/households with children of different ages. In the ICO-FACT project participants were recruited from families across income group, diverse geographic regions, and from families with a variety of household compositions (single-parent families, extended families and nuclear families), and both children, parents and grandparents were asked to participate. In Sweden, the UK and the US, an additional purposive sampling was made to recruit participants from minority and marginalized groups. The sampling strategy and data collected aimed for attentiveness towards pertinent local factors in each country, and with openness and sensitivity for individuals and varied life circumstances of potential participants.

### Participants and data collection

This study reports on data collected from 172 adolescents and young people aged 12–24 years in five countries: Chile, South Africa, Sweden, the United Kingdom (the UK) and the United States (the US). Data collected from adults are reported elsewhere. For an overview of participants and data collection, see Table I

**Table I.** Overview of the total number of participants in the ICO-FACT study and the number of adolescents and young people within respective country from which data and findings are reported in this study.

Country	Participants	Data collection period	Data collection methods
Chile	38 Families 38 adolescents (12–18 years old, 18 girls/20 boys)	June 2020–June 2021	Mulitmodal diaries via Indeemo AND Individual & family interviews
South Africa	64 participants 16 adolescents (11–18 years old, 10 girls/6 boys)	June 2020–March 2021	Mulitmodal diaries via WhatsApp AND Individual interviews
Sweden	117 participants 95 adolescents (14–19 years old, 55 girls/38 boys)*.	June 2020–June 2021	Written replies to open-ended questions (*N* = 66 children) OR Focus groups OR Individual interview
UK	73 participants 13 adolescents (12–18 years old, 8 girls/5 boys)	May 2020–June 2021	Mulitmodal diaries via Indeemo AND Individual & family interviews
US	59 participants 10 adolescents/young people (12–24 years old, 5 girls/5 boys)	May 2020–February 2021	Mulitmodal diaries via WhatsApp

Note: *Sex not reported by all participants.

Across the country settings, data were collected by either online app (Indeemo or whatsapp), via email or phone, or a combination of the above. In all cases, questions asked were similar and focused on how family life, social relationships, education, and everyday routines changed (or not) during COVID-19. Remote and/or digital methods via a mobile application or email was used across all contexts.

During the process, we found that we needed to add additional data gathering in consideration to national settings. In Sweden, face-to-face individual interviews or digital focus groups were conducted. Swedish adolescents who were not experiencing lockdown seemed less interested in reporting on their experiences via digital technology. In South Africa, additional telephone interviews were needed with adolescents as access to digital technology was low (Iqbal et al., 2023). The same questions were used for all settings, irrespective of data collection method. However, interviews allowed for questions to be repeated or rephrased, and for follow-up questions. The interviews lasted between 20 and 60 min.

### Data analysis

Data were analysed using thematic analysis (Sundler et al., 2019). Thematic analysis allows identifying, analysing and reporting of patterns of meanings, and to organize and describe meanings of the studied phenomenon. Initially, the analysis starts with carefully and repeatedly reading of the text to get an overall sense of characteristics and contents of the data. Thereafter, the analysis moves into a more systematic reading with focus on meanings. As different meanings and patterns of meanings are identified, these meanings are compared for differences and similarities while being condensed and arranged in initial themes. To grasp underling meanings and for a deeper understanding, reflections are needed on details of the data and its related meaning to allow new insights to emerge. As the process of analysis progresses, themes are possible to describe. Finally, the emerging themes and results need to be reviewed, discussed and further refined by the researchers.

The initial readings and understanding of data were made by each national research team without any translation or sharing of data between countries, in accordance to ethical considerations. This allowed for a first reading and preliminary understanding of meanings as expressed directly by the participants. A series of online meetings by authors across countries were held to discuss the data during analysis and in the development of this paper. As the analysis progressed, meanings were described in an English text to allow for comparison and description of patterns and meanings. Three of the researchers continued with the analysis and the search for and comparison of meanings from the descriptive text in English. Themes were derived and further developed from the text. The themes and the text were reviewed and refined by all authors. The researchers strived to remain sensitive to the voices of the adolescents and young people participating in this study in the process of analysis and writing of the results. Quotations have been selected by researchers from each country respectively to illustrate themes and meanings described in the results. The quotes used have been anonymized so as not to expose participants’ identities.

### Ethical considerations and data storage

This study complies with the ethical principles of The Declaration of Helsinki and follows ethical guidelines for each respective country and/or institutions. Data were collected with ethical approval in accordance with the regulations within each participating country and/or institution. In Sweden, the study was approved by the Swedish Ethical Review Authority (Dnr 2020–02155; Dnr 2020–04648), in the UK ethics approval was obtained from the IOE Research Ethics Committee (Z6364106/2020/04/132), IOE, UCL’s Faculty of Education and Society Research, University College London, in South Africa, ethics approval granted by the Faculty of Humanities Research Ethics Committee, University of Johannesburg (REC-01-084-2020), in US ethics approval was granted by the UCLA Institutional Review Board (IRB#20–000681), and in Chile the ethical procedures were approved by the respective committee of the University of Santiago de Chile.

Information about the study was given to all participants, and all participating adolescents and young people gave their informed consent to participate. Participants were given information that they could withdraw participation at any time without specifying reasons, and that withdrawal would not in any way affect them. In some countries, parents consented to their child’s participation in research, according to national ethical guidelines.

All data collected were stored securely at each site and handled by the national research team in accordance with the General Data Protection Regulation 2016 or national guidelines, and followed legislation and safety routines in each country and institution respectively.

## Results

**Figure 1.**
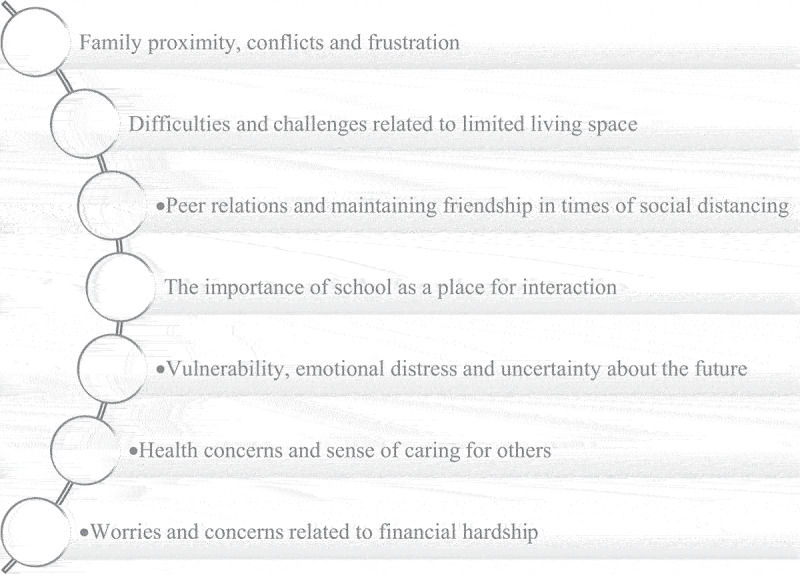
Overview of themes.

Experiences related to social relationships and health concerns of adolescents and young people are described in seven themes. See Figure 1.

These themes further describe experiences from the pandemic and the impact on individuals’ everyday lives, well-being and social relationships. Their experiences varied and were sometimes ambiguous including both negative emotions (e.g., feelings of loneliness, vulnerability, and emotional distress) and positive ones (e.g., related to experiences of increased togetherness with family). The adolescents’ reports express how everyday life and well-being became more affected by the consequences of the pandemic than the disease itself, as everyday life changed and worry for the future grew.

### Family proximity, conflicts and frustration

Adolescents and young people reported strains as well as gains with social distancing policies and stay at home orders: a stronger sense of togetherness but also more moments of conflicts, frustration, and irritation, as two sides of the same coin.

Overall, they pointed out that family proximity had benefited mental well-being and general happiness, and that time during stay-at-home orders was characterized by consideration for each other’s needs. Some individuals stressed in particular how stay-at- home orders and lockdowns had resulted in them spending more time with and feeling closer to their siblings. As a 17-year-old girl from the UK noted:

My relationship with my two brothers … , I think has changed, in that I do currently feel like our relationship is closer, especially with the younger, J, as we both do our school work at the same table everyday so we do mess around and where we usually argue a lot, and still do to be fair, we are closer now because of this. B is quite a withdrawn person so I do usually find it quite hard to feel like I have a close relationship with him, but during lockdown I feel like we have bonded and we are closer - he obviously trusts me because he let me cut his hair today.

Stay-at-home orders were linked to both positive and negative emotions. The following quote from a 19-year-old teenage girl from South Africa captures how she experienced life under lockdown with her family:

I feel like there’s two sides to this. I extremely love it and I’ve been enjoying it for the first few months and we got to learn new stuff about each other and get the time to talk about certain things that we never really had the time to talk about. But on the other side it is stressful because you don’t have time for yourself, it’s either one is barging in your room or the other is making noise. You know everyone must be in each other’s space and sometimes you’re like “oh my gosh please give me space” you know.

This quote illustrates two sides of young people’s experiences. On the one hand, social isolation resulted in better knowledge and understanding of each other, while, on the other hand, such experiences were also challenging and could result in frustration and conflicts. This illustrates the dynamic aspect of relationships and that they can change over time.

In Sweden, only a few individuals raised issues such as increased irritation between family members. Sweden was an exceptional case in that families did not experience any lockdown and most individuals attended preschool and school as usual. Social restrictions were in place, however, where young people had to be at home more as leisure activities were closed. Overall, adolescents’ and young people’s social relationships with family members changed, with new practices and ways of interacting evolving and forming everyday life.

### Difficulties and challenges related to limited living space

The lived space of everyday life was experienced as restricting and limiting as a consequence of stay-at-home orders during COVID-19. Adolescents and young people described how family life was structured in new ways with many parents working from home, and schools and preschools being closed. Some adolescents and young people had their parents at ordinary work and had to take up more responsibility at home. Young people and all family household members had to adapt to living in close quarters, and/or to new responsibilities at home (for example, in families where parents continued to work outside the home in “essential” jobs). Participants reported new or additional responsibilities, such as having to mind siblings and helping with daily chores. Older siblings described needing to take more responsibility for minding and/or doing schoolwork with younger sisters or brothers. As one Chilean 12-year-old boy reported:

[With the pandemic] I have to look after my sister more [aged 5]. At the beginning, we used to fight, but now I know how to calm her down when she starts to go crazy. I spend more time with my sister, and I have more time to understand her, and we do not fight as much.

As the above quote illustrates, changes in their living conditions, with increased responsibilities may have been hard at first. However, increased time with and responsibility for younger siblings could also lead to closer relationships.

Doing schoolwork at home presented many challenges, especially when sharing small spaces with others who were also working or doing schoolwork at home. Participants described how their concentration could easily be disturbed as there were so many other things that called for attention. They might be tempted to pick up their mobile phone or watch a streamed series rather than do their school work, in comparison to school where their actions would be more closely monitored. It was also difficult when the sphere of the home was shared with the family, as illustrated by a Swedish teenage girl:

There are a lot of people at home … and we have a puppy running around that needs a lot of attention, so there’s a lot happening and it’s very hard to concentrate.

The restricted lived space during social restrictions and lockdown also limited their social interaction with peers. The becoming aspect of life, as part of adolescents developing into early adulthood connected to peer relationships, that is, to learn and experience new things and social worlds outside the household, was experienced as being on hold for many of the adolescents and young people during the pandemic.

### Peer relations and maintaining friendship in times of social distancing

With social distancing restrictions, closed schools and lockdowns, peer contacts for adolescents and young people became primarily or only on-line, in different national contexts. Some individuals reported that it was difficult to interact with and interpret other people on-line compared to face-to-face. A 17-year-old UK girl reflected on how fleeting encounters at schools, exchanges of looks and nods, added to feelings of closeness among friends while “in lockdown, is kind of like you have to actually actively call them instead of just like saying hi in the corridor”. Thus, on-line spaces for interactions with peers required active effort and was experienced as reducing spontaneous connections. Participants in all nations brought up the advantages of being on-line for continued contacts with peers. However, on-line communication was mainly experienced as second-best, a “half-good” relationship. A quote from a 16-year-old South African boy illustrates this sentiment:

I would be able to chill with my friends before this; we would hang out after school. Now we just chat on Facebook and WhatsApp since I cannot meet them to tell them about my things and so on. It is only texting now. This corona is annoying.

In the UK, there were long periods of lockdowns and school closures. A 17-year-old girl reflected on how physical distance and on-line contacts impacted on her friendships and made peer relationships harder:

‘I think it’s a little hard to maintain friendships through screen and in comparison to real life. So, like I’m calling my friends and face-timing them instead of actually talking face to face which is like kind of frustrating as well, “cause the connection can, you know, be a bit slow like there’s always these problems and stuff so it’s a bit hard to maintain friendships.”

Some youth did not have access to digital devices, or to internet services, and consequently could not have on-line contacts with peers or continue with schoolwork as usual. As a 12-year-old boy in the UK from a low-income household stated: “I don’t really have WhatsApp and I don’t really have the other stuff as well”. Adolescents in Chile, especially adolescents from low socioeconomic status families, reported that social contacts with classmates decreased during the pandemic.

Peer relationships were described much in contrast with family life—as having stagnated or decreased rather than developed. That on-line communication enabled swift and “shallow” rather than deep/close contacts, was evident from their experiences, making it harder to maintain and form relationships.

### The importance of school as a place for interaction

Closed schools and preschools affected peer relationships and their thoughts about their future. Older youths reported worries about the future, as they were missing out on school. School transitions came across as being of particular concern for children in several countries; pandemic policies on social distancing made it harder to make new friends for children who changed schools or classes. For instance, Swedish adolescents in grade 9 (15- to 16-year-olds) disapproved of social events being cancelled or changed to on-line activities. They pointed out that physical meetings would have granted opportunities for them making new contacts and friends.

Younger adolescents in Sweden stated they were grateful that schools remained open. Adolescents from this national context regularly met peers face-to-face:

The friends I have attend the same school as I, most of them in the same class. Therefore, I spend time with them as usual [after school] since we would contaminate each other anyway at school.

As schools were open, they continued to meet peers and spend leisure activities together, as long as these were not cancelled, even though they did not hang out as much as before.

Distance/online schooling was seen as an advantage for those young people who for various reasons received home schooling prior to the pandemic, as stated by a 17 -year-old Swedish girl:

“I also think that there are very positive aspects of distance education … I know many ‘school refusers’ [with home-schooling prior the pandemic] that have been able to be a part of school in a whole other way and I think at distance education gives another possibility for people that can’t manage to come to school for various reasons.”

However, not all adolescent and young people experienced school or social media as safe social spaces. For instance, two British 12-year-old boys from different families expressed the relief they felt to be away from certain children who had been bullying them at school.

### Vulnerability, emotional distress and uncertainty about the future

Participants reported experiencing emotional distress and feeling lonely or sad at times. Physical peer contacts had decreased and appeared to have caused frustration, boredom, restlessness, sadness, and a sense of missing out on opportunities to meet new friends. Social isolation increased feelings of loneliness, as one Swedish 15-year-old girl said:

’You feel more down than usual since you are more on your own. You can’t see people all the time and you can’t hang out with everyone the way you’d like’.

In Sweden, adolescents reported they have been affected psychologically by the pandemic. Those whose mental health was fragile prior to the pandemic described how it became worse. They described preferably turning to friends and family for support. Seeking professional support felt awkward and difficult if one did not already have a contact such as a psychologist. If a contact had been established previously it was easier to get in touch with this person or health care services again. During the pandemic, some of the youths reported on difficulties to get help when needed for mental health problems, as an 18-year-old Swedish girl told us:

It’s harder now to talk to someone, if we think outside of the family … the school counsellor or youth clinic … both had drop-in times and everything [before the pandemic] … there was someone, I mean in a moment when I needed to talk to someone, there was always someone there before … now its difficult to find anyone, next to impossible.

Talking about emotional distress was difficult. On-line forums made it possible to pretend that everything was alright, and it was tricky to discover those in need of emotional support, as a 19-year-old Swedish boy stated:

This is tough, when you don’t have face to face contact in the way, because over the telephone or via text it’s hard to see the … ah … small hints … if they have prepared themselves before the conversation with me, to not show anything or … it’s easier to miss that now when we don’t have the same … ah … what should I call it … contact.

Exposing personal feelings on-line and in a group was difficult. Older adolescents experienced taking a greater responsibility during the pandemic, based on a sense of care for their family. They stated they felt as being those who “sacrifice the most’ during one of the most important parts of life when transitioning from childhood to adulthood.

Young people also expressed concerns and uncertainty related to the future. For instance, they described feeling uncertain when thinking about their graduation and their chance of getting a job and creating a career.

I was supposed to get a summer job but wasn’t able to work because of corona. Am I going to graduate without any work experience at all? Lots of people are unemployed … many young people get jobs in the cafés and restaurants, but will the job market be left? (girl, 18-year-old, Sweden).

Uncertainty about the future, together with feelings of loneliness, left some feeling depressed. In addition, the pandemic exacerbated worries and concerns young people already had about climate change and political instability.

Health concerns were also reported in relation to sports and leisure activities. In Sweden, where many, but not all, gyms and sport halls continued to be open, some avoided these, and others felt it as important with physical activities as usual for their well-being and to avoid feeling depressed. There was evidence of young people balancing different kinds of risks, as illustrated by a 17-year-old boy who ultimately chose to attend the gym despite worries about the COVID-19 infection: “I also get down when I don’t train so that’s a problem these days”. Commonly, leisure activities were chosen at a time when less people would be there, to avoid contamination.

### Health concerns and sense of caring for others

Changes in living conditions and family togetherness seemed to be closely related to a sense of caring. Adolescents and young people across countries expressed worries about their parents, siblings or other family members. For instance, they raised concerns about the vulnerability of grandparents, and how living together made it hard to protect elderly relatives with social distancing measures. This was especially the case in households where parents continued to work outside the home, for example as health or food workers, as in one-third of the U.S. sample. In other countries, such as Sweden where compulsory education continued physically at schools, being restrictive with social contacts and keeping distance towards others was quite impossible. There were individuals that criticized the policy of open schools, as one 15-year-old (gender not reported) stated:

“We the pupils can infect vulnerable groups with corona, and many can die. We may not be harmed by the virus, but we may infect others. Close everything down, save lives and get rid of corona”.

This quote illustrates how some individuals experienced that national COVID-19 guidelines restrained them from acting upon their sense of care and concern for their family. Such concern could wane over time, however, as a 17-year-old Swedish girl reflected:

“You’re not as careful as you were in the beginning”.

Participants also reported concern for peers. Adults encouraged young people to keep an eye on and stay in touch with friends who seemed to be in need of help. Young people described positive initiatives they took such as sending text messages to friends just to ask how they were feeling. But participants reported that it was difficult to keep an eye on friends and how they were feeling, as most meetings were online. Some friends talked about their feelings when they felt poorly, and some did not.

### Worries and concerns related to financial hardship

Scarce resources and cramped housing affected individuals across countries during the pandemic. Financial hardship was linked to frustration and worries. For instance, some of the South African participants lived in informal townships, where space both inside and around the home was limited. Also, many Chilean families were struggling financially due to unemployment. Adolescents from low-income families in particular raised concerns about financial difficulties. As a 12-year-old boy from the UK wrote early on in the pandemic:

We are worried about money in our family because my mum does not get enough benefit and she does not work, yet it is worrying and we at times ration what we have.

Financial straits could add more stress and conflicts, which impacted everyday well-being. Some adolescents were faced with harsh realities around poverty and loss as a result of the pandemic and witnessing their parents struggle.

## Discussion

Adolescents’ and young people’s experience indicate that their everyday life and well-being was affected by the *consequences* of the pandemic more than the disease itself. The pandemic seemed to make adolescents more impacted by the restrictions, making them vulnerable when being in a development period of life when peer interactions, as well as dreams and hopes for future, are vital for development. Our findings stress the intimate relationship between young people’s emotional well-being and social relationships. Social restrictions made children and young people vulnerable to feelings of loneliness and emotional distress. They felt that their future was now negatively affected and that they were the group in society who have sacrificed the most during and because of the pandemic.

The findings of the present study support previous work showing that adolescents and young peoples reported more or less positive experiences, as well as contrasting consequences of pandemic restrictions (Kerekes et al., 2021; McKinlay et al., 2022; Panarese & Azzarita, 2021). Waboso et al. (2022), similarly found children struggling during the pandemic, at the same time as they noted positive feelings and the development of coping strategies. The current findings show that relationships with family members were connected to positive change to a large extent, with an increased sense of caring and connectedness. The findings suggest that children value their families and time spent with family when their lived space was restricted. This was evident across all national contexts.

This paper focused on adolescents and young people’s social relationships and health concerns. Social restrictions during the pandemic made them vulnerable to feelings of loneliness and emotional distress, which impacted on their health and well-being. Their experiences of health were clearly “disturbed” by the pandemic, and their experiences point underscore that health and well-being are multifaceted and related to both social and bodily being (Gadamer, 1993/1996; Merleau-Ponty, 2002). In this study, adolescents’ and young people’s voices contribute to the understanding of their perspectives.

Social relationships affect our everyday life and well-being. Challenges related to peers were described by adolescents and young people, as well as health concerns and emotional distress when experiencing changes to social relationships and missing social contact outside the home during lockdown, similar to the findings of a previous study (McKinlay et al., 2022). In contrast, adolescents and young people also reported new forms of closeness with parents and siblings. As the pandemic continued, they seemed to make meaningful insights from their experiences of being isolated and restricted, with both pros and cons. There were, however, individuals struggling with their mental health during the pandemic and those worrying about the future.

Adolescents and young people raised concerns related to their educational progress, similar to the findings of Haffejee et al. (2022). Educational achievement and schools are important for the well-being of children and adolescents (Jiao et al., 2020; Lee, 2020). Education and health are fundamental human rights and play an important role in the achievement of Agenda 2030 goals. Everyone—including all children and adolescents, has the right to education and the highest attainable standard of health, even though a life free of illness and disease cannot be guaranteed. The health consequences of the pandemic on the health and well-being of adolescents and young people in the long term are still not known, and we do not yet know how or which individuals will be most affected. Even though we know that the pandemic has affected their education and social relationships, it may take years before we know *how* these were affected and the consequences.

The findings describe how adolescents and young people across countries, with some exception for participants from Sweden, had little to no face-to-face interaction with peers during long periods of stay-at-home orders and lockdowns. Decreased face-to-face peer contact has greatly impacted on peer relationships and the full developmental effects may only be clear over a longer time span (Tomlinson et al., 2021). The present study does not contradict findings that suggest that on-line contacts, such as gaming, helped alleviate boredom, frustration, and the sense of isolation (Bengtsson et al., 2021), but shows that adolescents and young people in the current study did not experience on-line interaction as “enough” and could not fully replace ordinary face-to-face contacts. Meaningful social relationships involve corporeality and physical interaction in a lived space together with others that are different in intensity and meaning from virtual encounters. The importance of physical meetings is proven by participants’ reflections on stay at home orders where intensified proximity to family members caused an increased sense of connectedness, togetherness and knowledge about one another, as uncovered elsewhere (Levita et al., 2020; McKinlay et al., 2022; Panarese & Azzarita, 2021). Research has indicated great resilience in children during the pandemic (Kerekes et al., 2021). Perhaps intensified and deepened family relationships were a contributing aspect.

Adolescents and young people from low-income families raised concerns about financial difficulties and hardship. Financial and digital resources come across as being important in relation to whether they could or could not maintain friendships and contact with peers, as well as participate in education and school activities. Moreover, on-line communication is dependent on stable and fast internet connections, which at times, and for some individuals, was not available. This should be understood in a context in which there is significant variation in the quality of internet connection in different countries around the globe, and in the number and regularity of virtual classes delivered by schools (CIAE-Universidad de Chile, 2020). Our findings both echoes and contrasts with work by Dewa et al. (2021), showing that adolescents and young people from lower income families were not necessarily, but at times, most vulnerable and isolated during the pandemic. Consequently, there are individuals at increased risk of, for instance, social isolation, and school failure.

Detection of children and adolescents at risk for violence and maltreatment must be considered. To the best of the authors' knowledge, the adolescents and young people in the current study did not report on any violence and maltreatment, which may be a limitation. Such individuals may be even more exposed in times of lockdown and stay at home orders due to financial straits and conflicts related to families struggling with harsh realities and poverty. Although the children in our study did not report any such experiences, there are no guarantees that it did not exist. There are studies that suggest that domestic violence increased during the pandemic (Anderberg et al., 2021; Katz et al., 2021). A study from the United Kingdom shows that adolescents who felt close to their parents at the start of lockdown reported fewer mental health difficulties and lower emotional distress than those who felt distant (Cooper et al., 2021). However, we know little about the experiences of adolescents and young people with more tense relationships with their families, and how relationships develop in time of strains and challenges brought about by the pandemic.

### Strengths and limitations

The current study was initiated by ambitious researchers in different countries around the world. It allowed for varied data to be collected. However, different strategies were needed in different countries for the study to be applicable.

In the current study, the use of technology was experienced to both enable and restrict data collection. To allow for openness, additional data were collected in countries when allowed or when technology use was low. There may have been potential participants that declined when lacking the technology needed or when not being comfortable or motivated to use it. On the other hand, the use of technology facilitated data collection in several countries in times of lock downs and restrictions that otherwise would not have been possible.

Using on-line diaries versus interviews for data collection was related to both limitations and benefits. On-line diaries seemed suitable for adolescents and young people to use for self-reports on their experiences, allowing for multimodal expressions (such as via videos or photos); however, uptake and depth of these entries were variable. Interviews were sometimes used to make up for limited diary accounts and allowed for probing, but at the same time were more time consuming to preform and transcribe.

A strength of the study is the combination of data from adolescents and young people from various countries around the world, with often detailed and timely accounts over a long period of time during the heaviest periods of pandemic restrictions.

## Conclusions

The everyday life and well-being of adolescents and young people were restricted and affected by the consequences of the pandemic more than by the virus itself. These experiences had an impact on well-being and mental health, where some individuals were feeling more exposed and vulnerable to emotional distress and loneliness than others. Positive aspects of the pandemic were related to the strengthening of family bonds, feelings of togetherness and sense of caring. Thus, family and peer relationships could be protective and support a sense of togetherness and belonging. Hence, social relationships are important to provide emotional support. Support for adolescents and young people should be tailored accordingly around social and emotional concerns, and ultimately encourage health and well-being.
